# The association between serum ferritin and bilirubin with glycemic control among patients with type 2 diabetes mellitus

**DOI:** 10.25122/jml-2023-0136

**Published:** 2023-11

**Authors:** Reem Al Argan, Dania Alkhafaji, Abdulmohsen Al Elq, Waleed Albaker, Yasir Elamin, Abrar Alwaheed, Mohammad Zeeshan, Zainab AlElq, Malak Alkhalifa, Rana Al Mansour, Shada Alghamdi, Abdulelah Al Ghamdi, Fatema Ismaeel, Adnan Almarzouq, Fatma Zainuddin, Reem AlSulaiman

**Affiliations:** 1 Department of Internal Medicine, College of Medicine, Imam Abdulrahman Bin Faisal University, King Fahad Hospital of the University, Khobar, Saudi Arabia; 2 Department of Medical Education, College of Medicine, Imam Abdulrahman Bin Faisal University, Khobar, Saudi Arabia; 3 Department of Medical Allied Services, King Fahad Hospital of the University, Khobar, Saudi Arabia

**Keywords:** Type 2 diabetes mellitus, ferritin, total bilirubin, direct bilirubin, glycemic control, HbA1c, fasting blood glucose, T2DM - Type 2 diabetes mellitus, DM - Diabetes mellitus, FBG - Fasting blood glucose, HbA1c - Glycosylated hemoglobin, DPP-4 - Dipeptidyl peptidase-4, SGLT2 - Sodium glucose cotransporter 2, GLP-1 - Glucagon-like peptide 1, IQR - Interquartile range, WBC - White blood cells, MCV - Mean corpuscular volume, MCH - Mean corpuscular hemoglobin, MCHC - Mean corpuscular hemoglobin concentration, BUN - Blood urea nitrogen, eGFR - Estimated glomerular filtration rate, Na - Sodium, K - Potassium, CO2 - Carbon dioxide, GGTP - Gamma-glutamyl transpeptidase, AST - Aspartate aminotransferase, ALT - Alanine transaminase, ALP - Alkaline phosphatase, TIBC - Total iron-binding capacity

## Abstract

Previous evidence has shown an association between serum ferritin and bilirubin levels in the development of type 2 diabetes mellitus (T2DM) and glycemic control. However, the evidence is scarce in Saudi Arabia. In this study, we aimed to evaluate the association between serum ferritin and bilirubin levels with glycemic control in patients with T2DM. This was a cross-sectional study that involved 153 patients with T2DM recruited from outpatient diabetes clinics. Participants were categorized into two groups: well-controlled and uncontrolled T2DM, based on their glycemic status. We focused on comparing the iron profile and bilirubin levels between these two groups and examining the influence of antidiabetic medications on these parameters. A total of 153 patients with T2DM were included (58.2% women and 41.8% men). In both univariate and multivariate analyses, ferritin levels did not have a statistically significant association with glycemic control. However, patients with well-controlled T2DM had a significantly higher median level of total bilirubin and direct bilirubin than those with uncontrolled T2DM. Only direct bilirubin showed a statistically significant association with FBG less than 130 mg/dl and HbA1c level less than 7.0%. Ferritin level was not associated with glycemic control in patients with T2DM. On the other hand, direct bilirubin level was an independent predictor of better glycemic control. Monitoring direct bilirubin levels could aid in predicting glycemic control in T2DM and could be a potential target for developing antidiabetic medications.

## INTRODUCTION

Type 2 diabetes mellitus (T2DM) is the most common chronic metabolic and endocrine disease worldwide. The recent increase in the prevalence of diabetes mellitus (DM) in Saudi Arabia represents a major clinical and public health problem [1]. T2DM results from a combination of insulin resistance and impaired insulin secretion by ß pancreas cells. It is typically a multifactorial disease involving multiple genetic and environmental factors [2]. Factors such as zinc, bilirubin, and iron levels have been identified to be associated with the development of DM, glycemic control, and diabetic complications [3-6].

Iron is an important cofactor for fuel oxidation and electron transport but can lead to oxidative damage if not carefully regulated due to the generation of powerful oxidants such as hydroxyl radicles [7]. Studies have linked unregulated iron, particularly in conditions like hereditary hemochromatosis and post-transfusion iron overload, with an increased risk of pancreatic damage and DM development [8, 9]. This connection has been supported by studies showing improved glycemic control with reduced iron levels using chelating agents or phlebotomy [10, 11]. Recent research suggests that iron may influence T2DM development and exacerbate complications, even in patients without apparent iron overload [3, 4]. There is strong evidence that iron storage in such patients is correlated with insulin resistance, metabolic syndrome, and gestational diabetes mellitus [3].

Bilirubin is one of the end products of heme catabolism in circulation and was reported as a potent antioxidant [12]. In addition, it was found to be protective against the risk of cardiovascular disease in prospective studies [13]. To our knowledge, the relationship between serum ferritin and bilirubin with glycemic control of T2DM has not been studied in patients from Saudi Arabia or the Middle East. This study aimed to investigate the association between serum ferritin and bilirubin levels and glycemic control in T2DM patients. As a result, this could enable physicians to understand the association between serum ferritin and bilirubin and glycemic control. It also aimed to determine the impact of available antidiabetic medications on these parameters. Understanding these relationships may help predict glycemic control in T2DM and facilitate the development of targeted medications. The findings could guide physicians in optimizing patient management strategies.

## MATERIAL AND METHODS

This cross-sectional prospective study was conducted from 1 February 2020 to 31 January 2021 and included 153 adult patients with T2DM. Patients were recruited from outpatient diabetes clinics of King Fahad Hospital of the University in Khobar, Eastern Province of Saudi Arabia. Inclusion criteria were adults (≥18 years) diagnosed with T2DM according to the American Diabetes Association 2020 criteria. The study excluded patients on iron supplements or anemia treatment in the past three months, those with a recent history of blood donation or transfusion, pregnant women, individuals with gastrointestinal malabsorption, bleeding disorders, acute illness or medications affecting serum ferritin levels, and patients with type 1 diabetes mellitus or chronic/end-stage kidney disease requiring erythropoietin.

### Data collection

The following data were collected:

1. Baseline data covered age and gender.
2. Medical history: age at T2DM diagnosis, comorbidities, duration of T2DM, complications and antidiabetic medications.
3. Laboratory tests: complete blood count, renal and liver profiles, iron level, total iron binding capacity (TIBC), ferritin, total bilirubin, direct bilirubin levels, fasting blood glucose (FBG), and glycosylated hemoglobin HbA1c.

Ferritin levels were measured using Chemiluminescent Microparticle Immunoassay technology, and bilirubin levels were determined by the Diazo Salt method at our laboratory. Participants were divided into well-controlled T2DM (HbA1c≤7.0%) and uncontrolled T2DM (HbA1c>7.0%) groups for comparison of iron profiles and bilirubin levels. After that, we conducted a multivariate analysis to evaluate the association of serum ferritin and bilirubin levels with glycemic control to exclude the effect of other cofounders. Finally, we analyzed the impact of different classes of anti-diabetic medications on ferritin, total, and direct bilirubin levels.

### Statistical analysis

Data was analyzed using IBM SPSS.26. All categorical variables were presented as frequencies and percentages, while all continuous data were presented as medians and interquartile ranges (IQR). The chi-square test or Fisher’s exact test was used to assess the association between variables. The Kruskal-Wallis test was used to compare the medians. Odds ratios (ORs) with their 95% confidence intervals (CI) were measured in multivariate analysis. Statistical significance was set at p<0.05. The sample size was calculated based on an estimated correlation coefficient (r) of 0.278 between serum ferritin and HbA1c, with a significance level (α) of 0.05 and a power of test (1-β) of 0.1, resulting in a minimum required sample size of 132 T2DM cases [14].

## RESULTS

### Participants' demographics and comorbidities

A total of 153 patients with T2DM were included in the study. Out of them, 26 patients (17%) had controlled DM, and 127 patients (83%) had uncontrolled DM. The study cohort was composed of 58.2% women and 41.8% men, with the majority (55.6%) being in the age range of 41-60 years, followed by the 61-80 years age group (36.6%). Dyslipidemia was the most common comorbidity found in 69.3% of cases, followed by hypertension in 55.6% of the cases. Furthermore, 27.5% of the patients had chronic liver disease, and 22.2% were diagnosed with non-alcoholic steatohepatitis. Chronic liver disease and non-alcoholic steatohepatitis were significantly associated with uncontrolled T2DM (p=0.05 and 0.047), respectively (Table 1).

**Table 1:** Participants demographics (n=153)

		Total	Well-controlled n=26	Uncontrolled n=127	p-value
**Gender**	Male	64	9 (34.6%)	55 (43.3%)	0.413
	Female	89	17 (65.4%)	72 (56.7%)	
**Nationality**	Non-Saudi	19	1 (3.8%)	18 (14.2%)	0.15
	Saudi	134	25 (96.2%)	109 (85.8%)	
**Age** **(Years)**	20-40	10	1 (3.8%)	9 (7.1%)	0.85
	41-60	85	15 (57.7%)	70 (55.1%)	
	61-80	56	10 (38.5%)	46 (36.2%)	
	>80	2	0 (0%)	2 (1.6%)	
**Comorbidities**	Hypertension	85	18 (69.2%)	67 (52.8%)	0.13
	Dyslipidemia	106	20 (76.9%)	86 (67.7%)	0.35
	Chronic Liver Disease	42	3 (11.5%)	39 (30.7%)	0.05*
	Non-Alcoholic Steatohepatitis	34	2 (7.7%)	32 (25.2%)	0.047*

Abbreviation: n: number; *Significant p-values are shown in bold

### Diabetes mellitus-related data

Most participants had a prolonged duration of T2DM, with 48.3% having the condition for 16-20 years or more. Among diabetes complications, diabetic retinopathy was the most frequent microvascular complication (38.56%), while coronary artery disease was the most frequent macrovascular complication (13.07%) (Table 2).

**Table 2:** Diabetes mellitus-related data

Variables		Frequency	Percentage
**Duration of diabetes (Years)**	<5	23	15
	5-10	25	16.4
	11-15	31	20.3
	16-20	38	24.8
	>20	36	23.5
**Complications**	**Microvascular complications**		
	Diabetic neuropathy	27	17.65
	Diabetic kidney disease	18	11.76
	Diabetic retinopathy	59	38.56
	**Macrovascular complications**		
	Coronary artery disease	20	13.07
	Stroke	7	4.58
	Peripheral vascular disease	3	1.96
**Medications**	Metformin	130	84.97
	DPP4 Inhibitors	65	42.48
	SGLT2 Inhibitors	65	42.48
	GLP1 Agonist	20	13.07
	Sulfonylurea	17	11.11
	Insulin Glargine	59	38.56
	Insulin Aspart	31	20.26
	Insulin Degludec	4	2.61

Abbreviations: DPP4: Dipeptidylpeptidase-4, SGLT2: Sodium glucose cotransporter-2, GLP1: Glucagon-like peptide 1.

In terms of antidiabetic medication use, 84.97% of patients used metformin, while 42.48% of patients used Dipeptidyldiptidase-4 Inhibitors and Sodium Glucose Co-transporter-2 Inhibitors. Finally, 61.43% of participants were on insulin therapy (Table 2).

### Comparison of laboratory parameters between well-controlled and uncontrolled T2DM

The median level of total bilirubin was significantly higher in patients with well-controlled T2DM compared to uncontrolled T2DM patients despite being in the physiological range (0.6 *vs* 0.4 mg/dl) (p=0.04). Likewise, direct bilirubin was significantly higher in patients with well-controlled T2DM compared to uncontrolled T2DM patients (0.28 *vs* 0.19 mg/dl) (p=0.015). On the other hand, iron evaluations, including iron, TIBC, and ferritin levels, were statistically insignificant between the two study groups (p>0.05). Additionally, other laboratory parameters were similar between the study groups except for chloride level, which was higher in the group of well-controlled T2DM (p=0.048) (Table 3).

**Table 3:** Comparison of laboratory parameters between well and uncontrolled T2DM (univariate analysis)

	Type II Diabetes - Median (IQR)			p-value
	Total	Well-controlled n=26	Uncontrolled n=127	
**WBC**	7.6 (6.05-9.05)	7.8 (7.1-11.1)	7.5 (6-9)	0.093
**Hemoglobin**	13.2 (12.05-14.3)	13.2 (10.9-14.1)	13.2 (12.1-14.3)	0.28
**Platelets** Mean (±SD)	252.6 (±82.5)	286.5 (±104.7)	246.2 (±76.3)	0.083^†^
**MCV**	86.6 (78.8-91.3)	86.8 (82.7-91.1)	86.1 (78.5-91.4)	0.651
**MCH**	27.4 (25.15-29.1)	27.5 (25.7-28.8)	27.4 (25.2-29.1)	0.927
**MCHC**	31.4 (30.8-32.2)	31.4 (30.8-31.9)	31.5 (30.8-32.3)	0.482
**BUN**	13 (10-16)	12.5 (9.5-16.5)	13 (11-16.5)	0.661
**Creatinine**	0.79 (0.69-0.91)	0.8 (0.7-0.9)	0.8 (0.7-0.9)	0.55
**eGFR**	90 (79-103)	84 (74.6-102.2)	91.7 (79.9-102.8)	0.322
**Na**	139 (137-140)	139 (137-140.5)	139 (137-140)	0.902
**K**	4.5 (4.2-4.7)	4.6 (4.2-4.7)	4.5 (4.2-4.7)	0.88
**Chloride**	103 (102-105)	104.5 (102-106)	103 (101-104.5)	0.048*
**CO2**	26 (24-28)	27 (25-28)	26 (24-27)	0.075
**Total Bilirubin**	0.5 (0.3-0.6)	0.6 (0.4-0.8)	0.4 (0.3-0.6)	0.04*
**Direct Bilirubin**	0.2 (0.1-0.3)	0.28 (0.2-0.3)	0.19 (0.1-0.2)	0.015*
**Albumin**	4.2 (3.9-4.4)	4.2 (3.9-4.4)	4.1 (3.9-4.4)	0.585
**AST**	18.5 (15-23)	21 (17-24.5)	18 (15-23)	0.088
**ALT**	21 (15-30)	21 (17-29.5)	20 (15-30)	0.493
**ALP**	76 (61.5-94)	72.5 (55-83)	78 (63.5-95)	0.2
**GGTP**	27 (18.5-43.5)	21 (17-31)	27 (20-45)	0.124
**Iron level** Mean (±SD)	63.9 (±26.1)	66 (±25.4)	63.4 (±26.3)	0.65^†^
**TIBC**	305.5 (272-340)	311 (294-332)	298 (268-340)	0.205
**Transferrin Saturation** (Iron/TIBC X 100)	20.9 (14.9-18.4)	19.9 (16.2-26.9)	21.5 (14.4-28.7)	0.65
**Ferritin**	74 (20-106.8)	70 (22.7-116.9)	75.1 (17.9-106.3)	0.7

Abbreviations: T2DM: Type 2 diabetes mellitus, IQR: Interquartile range, n: number, WBCs: White blood cells, MCV: Mean corpuscular volume, MCH: Mean corpuscular hemoglobin, MCHC: Mean corpuscular hemoglobin concentration, BUN: Blood urea nitrogen, eGFR: Estimated glomerular filtration rate, Na: Sodium, K: Potassium, CO2:Carbon dioxide, GGTP: gamma-glutamyl transpeptidase, AST: Aspartate aminotransferase, ALT: Alanine transaminase, ALP: Alkaline phosphatase, TIBC: Total iron-binding capacity. ^†^By t-test.

* Significant p-values are shown in bold.

### Association of serum bilirubin level with glycemic control after excluding patients with chronic liver disease and steatohepatitis (univariate analysis)

A univariate analysis after excluding patients with chronic liver disease and steatohepatitis showed a significantly higher median level of total bilirubin in patients with well-controlled T2DM compared to those with uncontrolled T2DM patients (0.55 *vs* 0.4) (p=0.025). Similarly, direct bilirubin was significantly higher in patients with well-controlled T2DM compared to uncontrolled T2DM patients (0.28 *vs* 0.19) (p=0.015) (Table 4).

**Table 4:** Association between serum bilirubin level and glycemic control after excluding patients with chronic liver disease and steatohepatitis (univariate analysis)

Variable	Univariate analysis		
	Well-controlled T2DM n=26	Uncontrolled T2DM n=127	p-value
**Total Bilirubin**	0.55 (0.4-0.8)	0.4 (0.3-0.6)	0.025*
**Direct Bilirubin**	0.28 (0.2-0.3)	0.19 (0.1-0.2)	0.015*

Abbreviation: n: number; *Significant p-values are shown in bold

### Multivariate regression analysis of the association between serum ferritin and bilirubin levels with glycemic control

Our multivariate analysis showed that male gender had significantly lower odds of better FBG (OR 0.29; p=0.002). On the other hand, direct bilirubin was an independent predictor of lower FBG less than 130 mg/dl (OR 14.18; p=0.038) and HbA1c level less than 7.0 % (OR 10.96; p=0.016). However, serum ferritin did not have a statistically significant association with glycemic control (p>0.05) (Table 5).

**Table 5:** Association of serum ferritin and bilirubin level with glycemic control (multivariate analysis)

Variable	FBG (Cutoff less than 130 mg/dl)		HbA1c (Cut off less than 7%)	
	Odds Ratio (Confidence Interval)	p-value	Odds Ratio (Confidence Interval)	p-value
**Age**	0.72 (0.38-1.34)	0.298	0.94 (0.43-2.09)	0.888
**Gender (Male)**	0.29 (0.13-0.63)	0.002*	0.77 (0.29-2.01)	0.59
**Chronic liver disease**	1.02 (0.4-2.59)	0.963	0.47 (0.12-1.88)	0.283
**Nonalcoholic steatohepatitis**	1.44 (0.54-3.87)	0.466	0.36 (0.07-1.88)	0.227
**Ferritin**	0.998 (0.93-1)	0.323	1 (0.93-1)	0.743
**Total Bilirubin**	0.31 (0.03-2.93)	0.309	1.47 (0.53-4.12)	0.46
**Direct Bilirubin**	14.18 (1.04-555)	0.038*	10.96 (2.35-450.63)	0.016*

Abbreviations: FBG: Fasting blood glucose, HbA1c: Hemoglobin A1C. *Significant P-values are shown in bold.

### Correlation between antidiabetic medications with ferritin and serum bilirubin levels

As seen in Table 6, metformin was associated with a lower bilirubin level, but it did not reach a statistically significant value (p=0.06), which could be due to the small sample size. Other antidiabetic medications were not significantly correlated with serum ferritin or bilirubin (p> 0.05).

**Table 6:** Correlation between antidiabetic medications with serum ferritin and bilirubin levels

Class of Medication		Median (IQR)					
		Ferritin level	p-value	Total Bilirubin	p-value	Direct Bilirubin	p-value
**Metformin**	Yes	51.76 (19.37-104.08)	0.373	0.4 (0.3-0.6)	0.293	0.2 (0.1-0.2)	0.06
	No	61.19 (23.96-178.16)		0.5 (0.4-0.6)		0.2 (0.2-0.25)	
**DPP4 Inhibitors**	Yes	51 (19.37-92.26)	0.349	0.5 (0.3-0.6)	0.908	0.2 (0.1-0.3)	0.613
	No	53.57 (20.9-125)		0.45 (0.4-0.6)		0.2 (0.1-0.2)	
**SGLT2 inhibitors**	Yes	53.04 (13.05-98.3)	0.356	0.5 (0.3-0.7)	0.718	0.2 (0.1-0.3)	0.859
	No	53.04 (22.64-116.69)		0.4 (0.4-0.6)		0.2 (0.1-0.2)	
**GLP1 agonist**	Yes	43.39 (25.81-102.5)	0.756	0.4 (0.3-0.6)	0.531	0.2 (0.1-0.2)	0.591
	No	54.32 (17.94-110.03)		0.5 (0.4-0.6)		0.2 (0.1-0.2)	
**Sulfonylurea**	Yes	55.11 (8.21-111.54)	0.575	0.5 (0.4-0.6)	0.474	0.2 (0.1-0.2)	0.209
	No	52.51 (20.9-106.81)		0.45 (0.3-0.6)		0.2 (0.1-0.3)	
**Insulin Glargine**	Yes	36.25 (14.85-138.04)	0.804	0.4 (0.3-0.6)	0.098	0.2 (0.1-0.2)	0.686
	No	55.66 (24.3-101.97)		0.5 (0.4-0.7)		0.2 (0.1-0.3)	
**Insulin Aspart**	Yes	55.11 (17.94-177.94)	0.459	0.4 (0.35-0.6)	0.565	0.2 (0.11-0.2)	0.743
	No	52.51 (21-98.6)		0.5 (0.3-0.6)		0.2 (0.1-0.2)	
**Insulin Degludec**	Yes	67.51 (36.91-169.15)	0.565	0.6 (0.5-0.65)	0.299	0.25 (0.15-0.3)	0.478
	No	51.76 (19.95-110.03)		0.4 (0.3-0.6)		0.2 (0.1-0.2)	

Abbreviations: IQR: Interquartile range, DPP4: Dipeptidylpeptidase-4, SGLT2: Sodium-glucose cotransporter 2, GLP1: Glucagon-like peptide 1.

## DISCUSSION

This prospective cross-sectional study of 153 patients with T2DM investigated the association between serum ferritin and bilirubin levels and glycemic control. We found the following key results. First, ferritin level did not have a statistically significant association with glycemic control in both univariate and multivariate analyses. Second, the median levels of total and direct bilirubin were significantly higher in the well-controlled T2DM group compared to the uncontrolled T2DM group. Interestingly, only direct bilirubin showed a statistically significant association with better glycemic control in multivariate analysis. Therefore, direct bilirubin level was an independent predictor of lower FBG less than 130 mg/dl (OR 14.18; p=0.038) and HbA1c level less than 7.0% (OR 10.96; p=0.016). Furthermore, male gender had lower odds of better FBG (OR 0.29; p=0.002). Finally, different classes of available antidiabetic medications did not influence either serum ferritin or bilirubin levels.

Ferritin is the major iron storage protein, and measurement of serum ferritin can reflect the iron load in the body [15]. People with high ferritin levels were found to be at a higher risk of DM. In addition, a positive correlation was demonstrated in previous studies between serum ferritin and glycemic control in T2DM patients measured by FBG, fructosamine, or HbA1c [16-19]. Our study showed that serum ferritin was not associated with glycemic control in patients with T2DM. The evidence is controversial in this regard. For example, Mojiminiyi *et al*. reported no improvement in glycemic control in patients with lower iron stores [20].

Furthermore, Dinneen *et al*. reported no association between hyperferritinemia and poorly controlled DM. Moreover, elevated ferritin was observed only in newly diagnosed diabetic patients, indicating a state of inflammation rather than an increase in iron stores [21]. This evidence was supported by another study that reported no improvement in glycemic control in patients treated with deferoxamine [22]. Conversely, Ford and Cogswell examined the association between ferritin concentration and glucose tolerance status, glucose concentration, insulin, and glycated hemoglobin in 9,486 adults from the United States (US). They reported a significant correlation between elevated serum ferritin levels, increased T2DM risk, and impaired glycemic control [23]. Supplementary, a meta-analysis of 16 studies, including 13,612 T2DM patients and 220,078 controls, denoted an association between the risk of T2DM and elevated ferritin levels [24]. Another study by Abd El-Halim *et al*. reported a significant positive correlation between serum ferritin levels and glycemic control in diabetic patients [16]. A recent study by Tummalacharla *et al*. found high ferritin in patients with uncontrolled diabetes mellitus [25]. This controversy could be due to the heterogeneity in the sample size, gender, and age of the population.

Cross-sectional studies have indicated a negative association between bilirubin levels and diabetes risk factors, including hypertension and metabolic syndrome [26, 27]. Additionally, bilirubin has been reported to play a protective role in individuals with diabetes mellitus [28]. In a study of 15,800 participants from the US, higher total bilirubin was associated with a 26% reduction in the risk of DM, which was confirmed by a multivariate analysis [28]. Our results showed a protective effect of bilirubin within the physiological range on glycemic control of T2DM patients. We identified direct bilirubin as an independent predictor of lower FBG (OR 14.18; p=0.038) and HbA1c level (OR 10.96; p=0.016) in multivariate analysis. Similarly, previous evidence has shown comparable results. Erkus *et al*. studied 215 diabetic subjects and reported higher serum bilirubin in well-controlled compared to poorly controlled diabetic patients (1.26±0.13 mg/dl *vs* 0.92±0.1 mg/dl), (p<0.001) [29]. Additionally, serum bilirubin was significantly and inversely correlated with HbA1c (r=-0,724, p<0.001) in Pearson correlation analysis in the same study. [29]. These findings are consistent with results from another cross-sectional study conducted by Farasat *et al*. [30], which reported similar associations. In their study, bilirubin levels showed a significant inverse relationship with HbA1c (r = -0.70; p< 0.05), insulin levels, insulin resistance, total cholesterol, low-density lipoprotein (LDL), and triglycerides. Moreover, bilirubin level was an independent determinant for the mean amplitude of glucose variability in women in a study of 77 patients with T2DM [31].

Furthermore, evidence supporting the protective effect of bilirubin was reported in relation to other aspects of DM. In a prospective cohort study involving 523 patients with impaired fasting and glucose tolerance, it was observed that low levels of serum total bilirubin were significantly associated with an increased risk of developing T2DM [32]. Furthermore, multiple studies have reported a protective role of serum bilirubin against diabetes complications [33, 34]. For example, a meta-analysis by Zhu *et al*. included 132,240 subjects from 27 studies. A negative nonlinear association between bilirubin concentration and the risk of diabetic complications was identified (OR: 0.77, 95% CI: 0.73–0.81) [33]. Specifically, the complications included diabetic nephropathy, retinopathy, and neuropathy [33].

The protective effect of bilirubin can be explained by its antioxidant and anti-inflammatory properties [35]. Animal studies have shown that administration of bilirubin in islet graft models reduced the serum levels of inflammatory mediators, which resulted in improved glucose control, enhanced glucose tolerance in diabetic recipients, and restored insulin-producing ability of transplanted islets [36]. Furthermore, other studies found a direct beneficial effect of bilirubin on insulin sensitivity [37]. Likewise, bilirubin has been shown to inhibit free radical production, thus improving insulin sensitivity and preventing vascular endothelial activation [38].

Our results showed that men had lower odds of controlled FBG than women, although gender was not associated with better HbA1c levels. Similarly, previous evidence has shown a tendency for lower FBG in women despite a lower likelihood of reaching glycemic goals compared to men [39]. Finally, our study did not identify any significant impact of currently available antidiabetic medications on serum bilirubin levels. Most of the previous research on the influence of antidiabetic medications on liver profiles predominantly focused on patients with liver impairment, primarily assessing their safety in such populations [40]. As a result, these studies often emphasized the evaluation of transaminase levels rather than serum bilirubin [40].

In contrast, our study differed from previous research, as it specifically investigated the effects of these medications on serum bilirubin levels within the physiological range.

The main limitation of our study is the relatively small sample size, which may not fully represent the broader diabetic population. This was largely due to the constraints imposed by the COVID-19 pandemic, which significantly reduced patient visits to outpatient clinics, thereby limiting our sample pool. Additionally, our analysis of antidiabetic medications faced challenges. We did not account for the duration of medication use or the potential effects of switching between different medications. In addition, the role of multiple medications was not explored.

## CONCLUSION

Ferritin level was not associated with glycemic control in patients with T2DM. In addition, only direct bilirubin was significantly associated with better glycemic control in multivariate analysis. Consequently, direct bilirubin level was an independent predictor of better glycemic control reflected by FBG and HbA1c levels. Finally, different classes of available antidiabetic medications did not influence serum ferritin or bilirubin levels in our study. We would recommend further randomized controlled studies to confirm our results, which would aid in using serum bilirubin, primarily direct bilirubin, to predict the glycemic control of T2DM patients. Eventually, the development of antidiabetic medications that target bilirubin as a potential marker would be recommended.

## Personal thanks

The authors acknowledge Dr. Hussain A. Alkhalifa for helping with data collection.
